# Single-instrument intracorporeal ligation versus polymer clip closure of the appendiceal stump in laparoscopic appendectomy: a prospective randomized study

**DOI:** 10.1186/s12893-026-03622-8

**Published:** 2026-02-23

**Authors:** Sezgin Topuz, Ali İşler, Emrah Cengiz, Mehmet Fatih Yüzbaşıoğlu, Mehmet Buğra Bozan, İlhami Taner Kale

**Affiliations:** 1 Department of General Surgery, Kahramanmaraş Sütçü İmam University Faculty of Medicine, Avşar Campus, West Ring Road Blvd. No: 251/A, Onikisubat, Kahramanmaraş, 46050 Turkey; 2 Department of General Surgery, Elazığ Fethi Sekin City Hospital, University of Health Sciences Turkey, Elazığ, Turkey

**Keywords:** Appendix stump closure, Laparoscopic, Single instrument, Appendectomy, Surgical technique

## Abstract

**Purpose:**

In laparoscopic appendectomy, stabilization of the appendix is required during knot-tying for stump closure. In the literature, additional ports or instruments have been used to achieve this stabilization. In our study, we employed a technical variation of intracorporeal knot-tying for appendiceal stump closure. This technique has not previously been used for this purpose. It does not require additional ports or instruments. The aim of our study was to evaluate the impact of this knot-tying technique on operative time and safety.

**Materials and methods:**

This study was conducted in adult patients, who were prospectively randomized into two groups and subsequently underwent laparoscopic appendectomy. First group included patients with single-instrument intracorporeal ligation, whereas second group included patients with polymer clip closure. Neither group employed any instruments other than the standard laparoscopic instruments. The operation time of the patients, the time from the completion of the dissection to the removal of the specimen (closure time) and complications were compared.

**Results:**

A total of 68 patients received laparoscopic appendectomy during the present study. There were 39 patients (57.4%) in the clip group and 29 patients (42.6%) in the ligation group. Of the 68 patients included in the study, 25 were women (36.8%) and 43 were men (63.2%). The median age of the patients was 34 (IQR:24–44) years. The mean body mass index (BMI) of the patients was 27.4 ± 4.5. Three patients were diagnosed with perforated appendicitis. Histopathological examination revealed acute appendicitis in 41 patients (60,3%), phlegmonous appendicitis in 26 patients (38.2%), and a normal appendix in 1 patient. The median operative time was 19 (IQR 13–28) minutes in the clip group and 35(IQR 30–45) minutes in the ligation group. Additionally, the median closure time was 3(2–9) minutes and 7 (4–13) minutes, respectively. Operative and closure times were longer in the Ligation Group (both, *p* = 0.00001). In the clip group, three patients developed wound site infections and one patient developed intra-abdominal abscess. In the ligation group, two patients developed wound site infections and one patient developed intra-abdominal abscess. There was no difference in total complication rates. (*p* = 1)

**Conclusion:**

Closure using single-instrument ligation prolonged the procedure time compared to clip closure and resulted in similar complication rates. This technique, previously unused for closing the appendiceal stump, can be performed with standard laparoscopic instruments without requiring additional surgical instruments or incisions. The method employed could be regarded as a technique that ought to be included in a surgeon’s repertoire of skills for various clinical scenarios.

**Trial registration:**

Clinicaltrials.gov (NCT06443749), 29.05.2024.

**Supplementary Information:**

The online version contains supplementary material available at 10.1186/s12893-026-03622-8.

## Introduction

Acute appendicitis is one of the leading causes of surgical acute abdomen, and appendectomy is considered the main treatment modality. To prevent stump leakage and associated postappendectomy septic complications, it is critical to close the stump during appendectomy [1, 2]. Although stump leaks following appendectomy are reported as rare, the incidence increases in complicated cases. Taguchi and colleagues reported a leak rate of 12.5% after laparoscopic appendectomy in patients with complicated appendicitis [3]. Similarly, intra-abdominal abscess formation following appendectomy is considered a rare complication, with an incidence reported in the literature of approximately 2–3% [4]. However, given the high frequency with which appendectomy is performed, even this relatively low rate translates into a substantial number of cases, which may require additional interventions [5]. 

In open appendectomy, before removing the appendix, its base is ligated with absorbable material to close the appendiceal stump [6]. The use of minimally invasive surgery is increasing in all fields of surgery. Sahm et al. reported that the management of acute appendicitis has largely shifted toward laparoscopic appendectomy. In laparoscopic appendectomy, various techniques are employed for appendiceal stump closure [7]. Commonly used stump closure methods reported by previous studies are listed in Table 1.

**Table 1:** Commonly used stump closure methods reported by previous studies

Metal endoclip [8, 9]
Preassembled endoloop [10, 11]
Ligating and suture technique[12, 13]
Endostapler [14, 15]
Polymeric clip [16, 17]

Various techniques have also been described for ligation and suturing of the appendix’s root, including intra- or extracorporeal methods, ligation using a transabdominal sling suture[18, 19], using a knot pusher for ligation [20], and sliding endoscopic knots [21]. In 2022, Pogorelic and colleagues reported that clipless harmonic scalpel laparoscopic appendectomy is a safe and effective method for treating acute appendicitis in children [22]. 

The present study aimed to use a ligation method that, to the best of our knowledge, was previously unused for appendiceal stump ligation and compare it with the appendiceal stump closure method using a polymer clip.

## Materials and methods

Upon approval from the Kahramanmaraş Sütçü İmam University Medical Faculty Hospital ethics committee (Date: 25.10.2023/Decision No: 02), patients diagnosed with acute appendicitis and scheduled for laparoscopic appendectomy were prospectively included until March 2025. All patients were informed about the study, and written consent was obtained from each of them. All procedures performed in this study adhered strictly to the ethical standards of the Declaration of Helsinki. This study was conducted and reported in accordance with the CONSORT 2025 guidelines for randomized trials. These patients were assigned to two groups. This study was conducted in adult patients, who were prospectively randomized into two groups and subsequently underwent laparoscopic appendectomy. Randomization was performed using the coin toss method. Immediately prior to the initiation of the case, the surgeon was informed whether the clip or ligation method would be used. All randomized patients were analyzed according to the groups to which they were initially assigned, regardless of the actual treatment received. Group 1 included patients who would receive appendiceal stump closure using a polymer clip (i.e., Clip Group). Group 2 included patients who would receive closure with a single-instrument intracorporeal knot (i.e., Ligation Group). In this study, the clip group was designated as the standard of care arm, against which the ligation group was compared. A study by Özdemir et al. was taken as reference for operation time, and the effect size was calculated as 0.82. The G Power software estimated the required total sample size as 48 (24 in each group), with an alpha error and power of 0.05 and 0.80, respectively [20]. After group randomization was achieved, patients’ data—including age, sex, body mass index, previous abdominal surgeries, medications associated with bleeding diathesis, as well as additional complexities such as ileus, perforation, and peritonitis (based on imaging findings and surgical reports)—were systematically recorded. After laparoscopic dissection, the appendiceal stump was closed using a polymer clip (Ligation/Polymer clip XL, LOOKMED, Wujin Changzhou City, Chine) in the Clip Group, and with an intracorporeal knot tied using a single instrument and a 75 cm, size 0 absorbable suture in the Ligation Group. The operative times of the patients, time from dissection completion to pathologic piece removal from the skin (set as the closure time), postoperative complications (postoperative 1 month), including intra-abdominal abscess, hemorrhage, fistula, and pathological findings, were recorded and compared. Six months after the surgery date of the last patient enrolled in the study, all participants were re-contacted and evaluated for postoperative complications. Complications were classified using the Clavien-Dindo grading system [23]. The study was registered at ClinicalTrials.gov with the number NCT06443749.

All patients aged over 18 years with acute appendicitis who had no contraindications to laparoscopic appendectomy were included in the study. Pregnant patients with appendicitis, and patients with interval appendectomies and mucoceles were excluded from the study.

The primary outcome measure of the study was the comparison of operative time and closure time between the two groups. The secondary outcome measure was the comparison of postoperative complications.

### Surgical technique

**Fig. 1:**
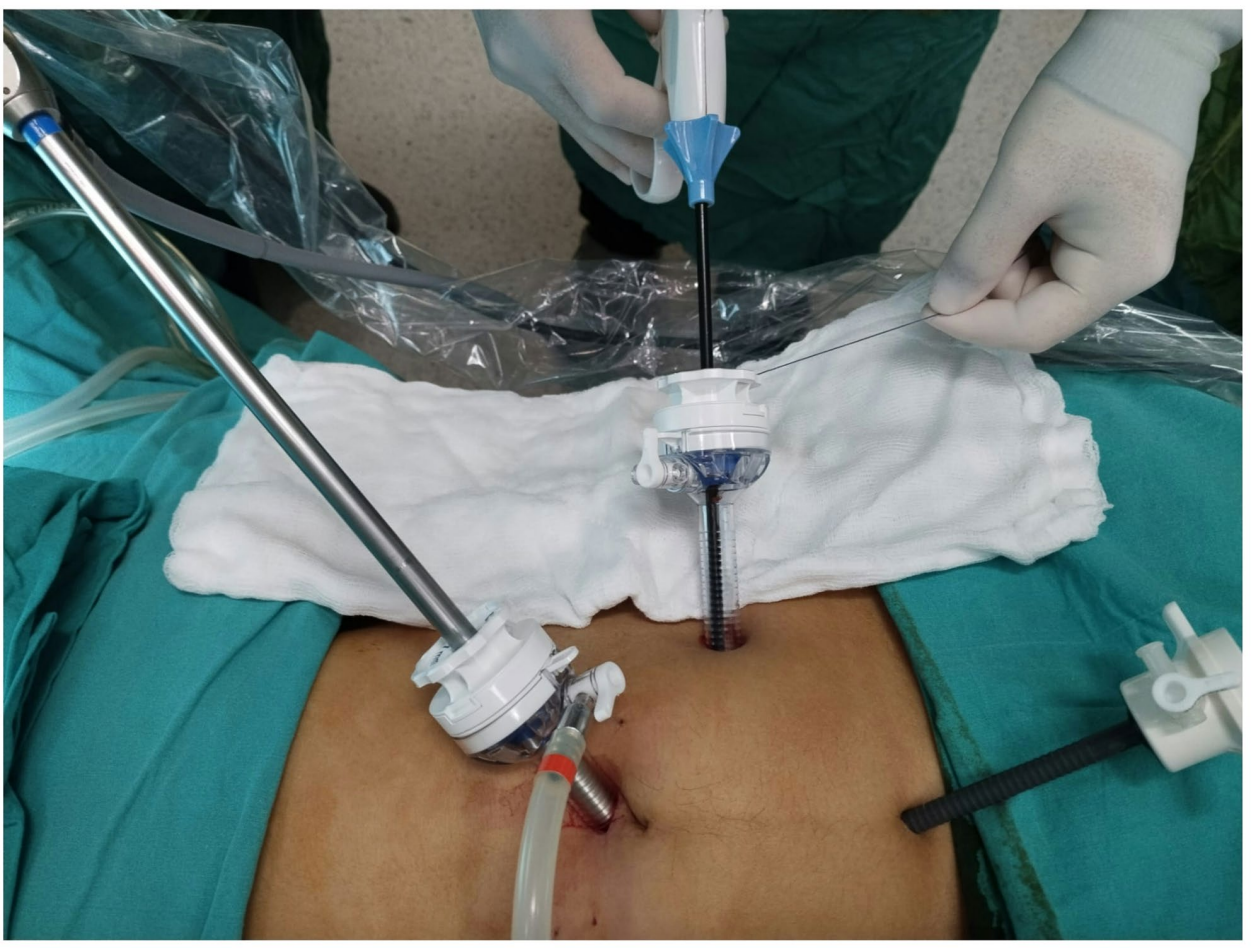
Trocar placement and the method by which the surgeon holds the suture extending out of the trocar in the left lower quadrant during ligation

**Fig. 2:**
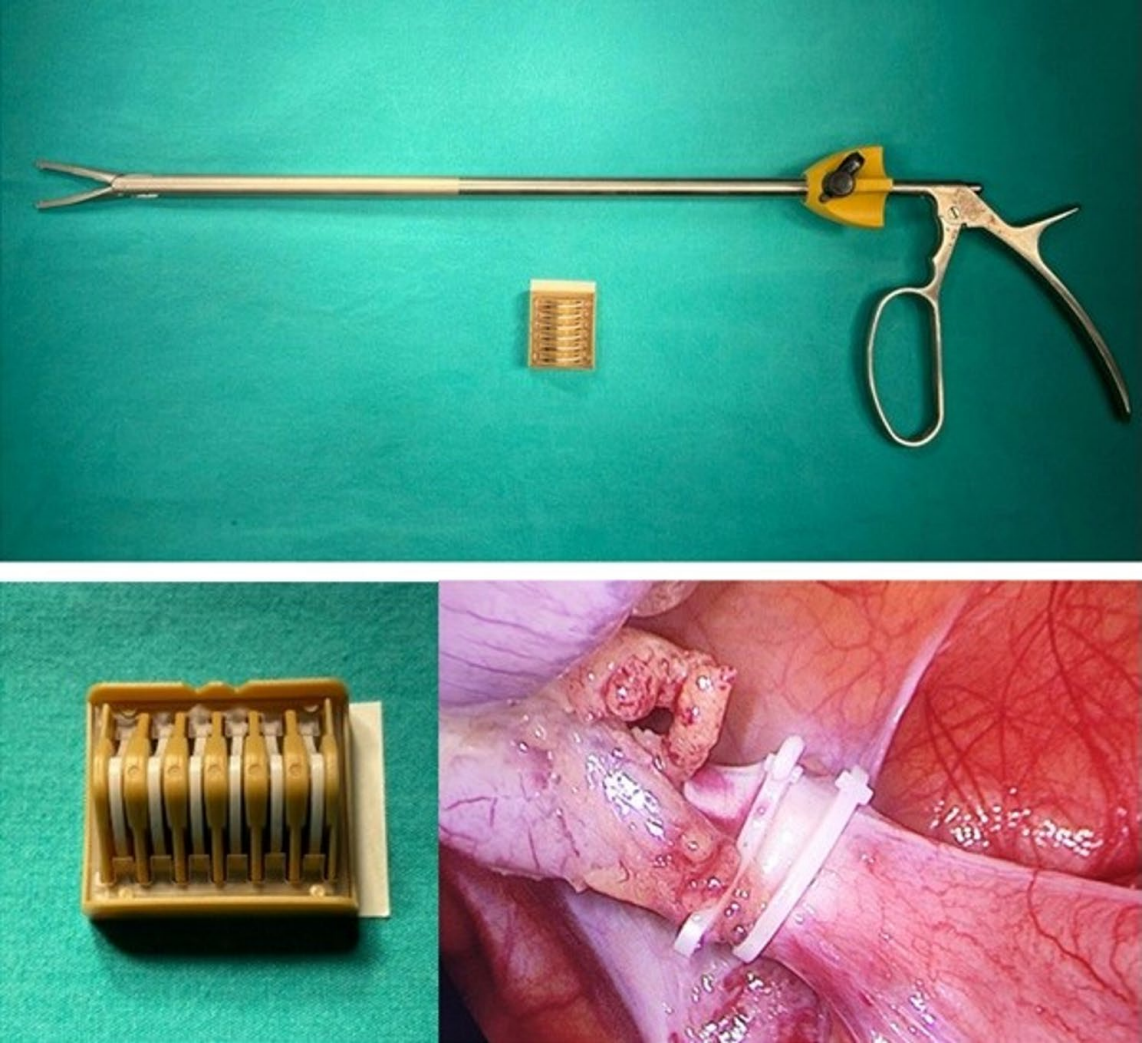
Clip applier (top), clip cartridge (bottom left), and intraoperative view of the appendix stump closed with clips (bottom right)

Patients with acute appendicitis were operated under general anesthesia, in the supine position, following skin antisepsis. All patients received prophylactic cefazolin IV (1 g) prior to the procedure. The abdominal cavity was entered through an approximately 1.5‑cm incision above the umbilicus using Hasson’s technique [24]. A 10 mm trocar was inserted at the site, and a pneumoperitoneum was established. This incision was used as the camera port. In all cases, 30° optics were used. Under 11 mmHg pressure, the procedure was performed in the Trendelenburg position by turning the operating table to the left, with a 10 mm trocar from the left lower quadrant and a 5 mm trocar over the pubis **(**Fig. 1). The mesoappendix was cut using a vessel sealing device and the base of the appendix was exposed. In the Clip Group, the appendix root was closed using a polymer XL clip (16 mm) and appendectomy was performed by cutting approximately 2–3 mm above the clip **(**Fig. 2). In the Ligation Group, the appendix was suspended using a grasper inserted through the trocar on the pubis. One end of a 75 cm, No. 0 absorbable suture (Polysorb™ (Covidien), composed of Lactomer™ (glycolide/lactide copolymer) was placed near the appendix in the abdomen using a laparoscopic dissector through the 10 mm trocar in the lower left (Fig. 1**).** The other end of the suture, which passed through the same trocar and remained outside, was held in the nondominant hand with slight tension. The suture held around the appendix using the tip of the dissector was pulled slightly, the appendix base was ligated by tying the knot at least three times, and the appendix was cut to complete the appendectomy (Video1). Where possible, the surgical specimen was removed directly from the trocar in the left lower quadrant. Otherwise, it was removed using an endobag inserted through the same trocar. The fascia at the trocar entry site above the umbilicus was sutured. The operations were terminated upon completion of skin sutures. All surgical procedures were performed by a single surgeon, who had 15 years of overall surgical experience and had been routinely performing laparoscopic suturing and ligation techniques for the past 5 years.

#### Video 1

Video demonstrating ligation with a single instrument during laparoscopic appendectomy (ligation group/Group 2). During the procedure, the appendix is stabilized using an instrument introduced through the suprapubic trocar, while the suture material and the dissector performing the knot are inserted via the left lower quadrant trocar.

### Statistical analysis

The statistical package for the social sciences (SPSS, IBM) version 20 was used for statistical analyses. For the purposes of the study, descriptive statistics were presented as mean ± standard deviation for continuous variables and frequency percentage for categorical variables. For data that do not follow a normal distribution, the median and IQR (Interquartile Range) values were provided. The normal distribution hypothesis for continuous variables was tested by Shapiro-Wilk test. For numerical data, the Student t test and Mann–Whitney U test were used when parametric test conditions were met and not met, respectively. For the analysis of categorical variables, the Chi-Squared Tests were used when appropriate, otherwise Fisher’s Exact Test was used. A p value < 0.05 was considered statistically significant.

## Results

A total of 68 patients received laparoscopic appendectomy during the study. There was no intraoperative transition to open surgery. In the Clip Group, the ligation method was used in two cases because the clips were insufficient to secure the base of the appendix. In the Ligation Group, the surgeon performed additional ligation in one patient and applied an extra clip over the ligation in four patients due to uncertainty regarding the primary method. **(**Table 2**)**

**Table 2:** Cases where the clip failed to clamp or the surgeon was not confident about the primary ligation

*n* = 68	Clip *n* = 39	Ligation *n* = 29
Failed to clamp	2 (5%)	0
Additional ligation or clip	0	5(17%)

Of the 68 patients included in the study, 25 were women (36.8%) and 43 were men (63.2%). The median age of the patients was 34 (IQR:24–44) years. The number of patients who received clip closure and ligation was 39 (57,4%) and 29 (42,6%), respectively. The descriptive characteristics of the patients are given in Table 3.

**Table 3:** Descriptive characteristics of the patients

*n* = 68		Median	IQR
Age		34	24–44
Body Mass Index (mean ± SD)		27,4	4,5
Length of Hospital Stay		2	1–2
		n	%
Sex	Male	43	63,2
	Female	25	36,8
Closure	Clip	39	57,4
	Ligation	29	42,6
Medication associated with bleeding diathesis(ASA)		4	5,9
Previous Surgical Incisions	None	58	85,3
	Med inf	1	1,5
	Caesarian	5	7,4
	Laparoscopy	4	5,9
Pathology	Acute appendicitis	41	60,3
	Acute phlegmonous appendicitis	26	38,2
	Vermiform appendix	1	1,5
Complicated Appendicitis	No	57	83,8
	Yes	11	16,2

Upon reviewing complications, none of the patients experienced bleeding, fistula, or hematoma during the 1-month postoperative follow-up. However, there was one case with abscess in each group, and open surgery was performed because percutaneous drainage was not feasible. Exploration performed during surgery revealed no leakage at the appendiceal stump. On postoperative day 3, the abdominal drains were removed, and the patients were discharged without any additional complications. Both patients were diagnosed with complicated appendicitis. In five patients, superficial surgical site infections occurred and were successfully managed with local wound care and antibiotic treatment. Six months after the date of the last appendectomy case, all patients were re-evaluated, and no newly developed complications were observed. Complications are given in Table 4. The Clavien-Dindo classification system was used to evaluate surgical complications. In our study, the complications observed in one patient from the clip group and one patient from the ligation group were classified as grade 3b. Additionally, superficial surgical site infections that developed in three patients in the clip group and two patients in the ligation group were classified as grade I [23]. The reoperation rate was 2.7% in the clip group and 4.1% in the ligation group. There was no significant difference in complication risk between the clip and ligation groups (RR: 0.991; 95% CI: 0.240–4.092).

**Table 4:** Complications

	Clip (*n*:39)	Ligation (*n*:29)
Abscess	1(%2,6)	1(%3,4)
Hematoma	0	0
Bleeding	0	0
Fistula	0	0
Wound site infection	3 (%7,7)	2 (%6,9)
Total complications	4(10,3%)	3(10,3%)
Reoperation (İAA)	1(2,6%)	1(3,4%)

*İAA *Intra-abdominal abscess

When comparing age, body mass index (BMI), and operative times between the stump closure methods, no statistically significant differences were found for age and BMI. However, operative and closure times were significantly longer in the Ligation Group. When comparing all complications, no statistically significant difference was found between the groups. Comparisons are given in Table 5.

**Table 5:** Comparison of groups

Variable		Clip(*n* = 39)	Ligation(*n* = 29)	HL (95% CI)	*p*
Age (Median (IQR)		36 (25–48)	33 (23–41)	–4 (−11to 2)	0,234**
OP (min) (Median (IQR)		19 (13–28)	35(30–45)	17 (13 to 20)	0,00001**
CT (min) (Median (IQR)		3 (2–4)	7 (5–8)	3 (2 to 4)	0,00001**
BMI (Mean ± SD)		27,4 ± 5,2	27,5 ± 3,7		0,952*
Pathology		n (%)	n (%)		
AA (n:42)		27(64,3%)	15(35,7%)		0,142***
APA (n:26)		12(46,2%)	14(53,8%)		
Prior Abdominal Surgery	No	33(84,6)	25(86,2%)		1,0****
	Yes	6(15,4%)	4(13,6%)		
Total Complications					
None (n:61)		35(57,4%)	26(42,6%)		1,0****
Yes (n:7)		4(57,1%)	3(42,9%)		

For BMI, the p-value was 0.676 in the clip group and 0.436 in the ligation group, indicating no significant deviation from normal distribution. Homogeneity of variances was evaluated using Levene’s test, yielding a p-value of 0.094

*AA* Acute appendicitis, *APA* Acute phlegmonous appendicitis, *HL* Hodges–Lehmann Estimator, *OP* Operative time, *CT* Closure time

*Independent Samples Test, ** Mann–Whitney U, ***Chi-Squared Tests, ****Fisher’s Exact Test. Normality was assessed using the Shapiro-Wilk test

## Discussion

The present study initially aimed to evaluate the use of a variation of the intracorporeal knotting technique, which had not previously been employed for ligation of the appendiceal stump. Compared with clip closure methods, as with all suturing techniques, both the operative time and the closure time were longer; nevertheless, the technique was applied in 24 cases without intraoperative complications and without the need for additional ligation or clip application.

Acute appendicitis is a prevalent condition, and the use of minimally invasive surgery for appendectomy has become widespread [7]. It is reasonable to assume that the ideal closure method in laparoscopic appendectomy should not prolong operative time, damage or loosen the appendix base, and should be compatible with laparoscopy, cost-effective, and widely available. However, there is an ongoing debate as to which method meets these requirements and is better than others [2]. 

Upon a review of the incidence of acute appendicitis across all age groups, half of the patients are < 30 years old and it occurs most prevalently in the second decade of one’s life [25]. The patients included in our study were aged over 18 years, comprising 43 men (63.2%) and 25 women (36.8%), with a median age of 34 (IQR: 24–44). Due to the use of a coin toss method for randomization, an unequal distribution occurred between groups, resulting in a higher number of patients in the clip group. The present study included two groups, Ligation and Clip Groups, and intergroup comparison based on age (*p* = 0,234) and BMI (*p* = 0,952) confirmed that they were statistically similar. None of the patients were diagnosed with malignancy upon examination of pathological findings. When one patient with negative appendectomy (Vermiform appendix) was included in the acute appendicitis group (for statistical analysis purposes), No significant intergroup difference was found regarding the pathological findings of phlegmonous and acute appendicitis (*p* = 0.291). Previous abdominal surgeries, which could potentially affect the operative time, were identified in a total of 10 patients. However, during the operation, no adhesions severe enough to complicate the procedure were observed in any of these patients. These findings suggest that the Clip and Ligation Groups had comparable baseline characteristics.

A meta-analysis by Najah et al. reported that intracorporeal suturing techniques required prolonged operation time [26]. In our study, operation time was statistically longer in the ligation group, consistent with the findings of the aforementioned meta-analysis (*p* = 0.00001). In some studies, no statistically significant difference was found between the operation times of closure with sutures and closure with clips [27]. Orhan et al. compared homolog clips with intracorporeal ligation but did not provide details about the intracorporeal ligation technique they used. These studies primarily focused on operation times [27]. However, operation time may be affected by multiple factors, including surgical adhesions, occurrence of perforation, and drain placement. Therefore, unlike the previous studies, the dissection time was excluded in the present study and the closure procedure time was calculated separately. Upon a comparison of operation time, the time spent for ligation was statistically significantly longer than that for clipping (*p* = 0.00001). However, the observed difference in closure time was 3 min, which was considered an acceptable duration (Hodges–Lehmann estimator for the median difference: 3.0; 95% CI: 2.0–4.0).

Upon a review of the total complications, there was no statistical difference between both groups (*p* = 1.0). One intra-abdominal abscess occurred in each group and was treated with open surgery because percutaneous drainage was not feasible. No leakage or problems were observed at the stump during intraoperative examination. Both patients were cases of complicated appendicitis. Therefore, in these two cases, postoperative abscess formation was due to complicated appendicitis rather than a problem related to the stump. Meta-analyses and prospective randomized studies have reported no significant difference between closure methods in terms of complications [26, 28, 29]. The findings of the present study are consistent with those reported in previous studies. However, while Gomes et al. suggested that the use of metal clips should be avoided in cases where the appendix base was necrotized, Najah et al. reported that endostaplers, which were likely to be effective in terms of complications, were used less frequently [1, 26]. Although we describe our technique as distinct from previously reported ligation methods, the core procedure remains intracorporeal knot tying. In the present study, the conventional intracorporeal knot was utilized, with the key distinction being its execution using a single instrument through a single trocar—without the need for additional devices or adjunctive maneuvers. The efficacy of conventional knot-tying techniques in terms of complication rates has been demonstrated in prior meta-analyses [30]. The lack of a significant difference in complication rates between groups in this study is consistent with previous research.

Different knot techniques with relative advantages and disadvantages are used in laparoscopic surgery. These techniques can be classified as intra- and extracorporeal. The intracorporeal technique is typically performed using two instruments [31] and it allows for ligation of the appendix root. However, for a comfortable ligation using this method, different techniques may be required to hold and stabilize the appendix. To overcome this problem, Ateş et al. performed the procedure by suspending the appendix to the abdominal wall with a number 1 silk using a flattened needle, entering the abdomen from the right lower quadrant. In their study, they compared titanium clips with intracorporeal knotting and included a total of 61 patients. In the knotting group, the appendix was stabilized by inserting a needle holder through the right lower quadrant and suspending the appendix. Intracorporeal ligation was performed using a two-instrument technique, which aimed to facilitate knot placement [19]. In the present study, a similar number of patients were evaluated, and no additional intervention was required. Sliding or surgeon’s knots are among the techniques used. Their disadvantages include the need for an additional knot pusher, and although it can be used in ductal structures, including the appendix, it is not preferred in sensitive tissues [31]. In the present study, a grasping device was used with the help of an available suprapubic trocar to grab and hang the appendix, the appendix was fixed for a comfortable ligation, and there was no need for an extra intervention technique or extra tools such as a knot pusher. In laparoscopic appendectomy, practical solutions for stump ligation can also be adapted from open surgical techniques. For example, Lima et al. performed extracorporeal appendectomy in patients with a body mass index below 27 by utilizing the umbilical trocar, achieving the procedure with lower cost, safety, and speed [32]. In relation to the subject of the present study, it can be considered as an example in which manual sensitivity could be utilized without the need for additional instruments or trocars.

It is foreseeable that overtightening the suture material in inflamed and fragile tissue during ligation of the appendix root may cause tissue damage, while insufficient tightening may fail to adequately close the lumen. Therefore, manual sensitivity is required during the ligation procedure. One of the disadvantages of laparoscopic surgery is the reduced tactile sensation, including tension and pressure in the tissue, because the instruments used to hold the tissue intervene between the surgeon and the tissue [33]. By holding one end of the suture externally and performing ligation without the use of any instruments other than gloves and the suture itself, intermediaries are eliminated, which may allow for improved tactile feedback. The present study also employs a technique in which manual sensation is preserved. To the best of our knowledge, the ligation method in question has not been previously used for ligating the appendix root. However, Thanakumar et al. described this knot in single incision laparoscopic surgery and used it safely in laparoscopic cholecystectomy [34]. Notwithstanding the above, their description is technical and not a part of a research article. The present study is the first prospective randomized study, which used this technique in appendectomy. Although this study is the first in which the technique has been used in laparoscopic appendectomy, it is a more realistic approach to consider it not as a new technique but rather as an innovative technical variation.

Polymer clips are widely used, and their reliability, rapid application, and relatively favorable cost have been highlighted in numerous studies [35, 36]. No cost analysis was performed for the purposes of this study. However, based on 2025 data from our hospital, the 0 absorbable suture we used was purchased for $2.1 per unit, whereas the price of a single cartridge of polymer clips was $11.9. This indicates that the clips are approximately five times more expensive. It should also be noted that this price does not include the reusable polymer clip applier, which was last purchased by our hospital for $200. Özdemir et al. reported that the use of sutures for ligation was much more cost-effective than the polymer clip [20]. In a study conducted in 2019, the treatment costs from hospital admission to discharge were compared, and no difference was found between homolog clips and intracorporeal ligation. However, the study did not provide details on how the cost analysis was performed [27]. In the Clip Group, a clip and an extra applicator to place the clip to the appendix root were needed. In addition to the cost issues, these polymer clips and applicators can be difficult to obtain. In contrast, the number 0 suture used in the Ligation Group is a standard material available in every operating room, making supply issues unlikely.

Although homologous clips are available in different sizes, they sometimes fail to clamp the tissue. The clip may fail to close or the clip’s tab may bite the tissue without grasping it, damaging the tissue, especially in tissues with a large lumen, e.g., a large appendix or cystic duct [34]. As additional data from our study, in two cases in the Clip Group, the stump was wide and ligation was performed because the clip could not clamp it. During the ligation process, the ligation of the tissue with appropriate strength as well as the surgeon’s experience and perceived safety were prioritized. For five cases in the Ligation Group, the surgeon used an additional clip even though there was actually no visible laxity. These findings suggest that ligation should be considered in cases with a large appendiceal root that cannot be accommodated by the clip. However, the surgeon might have concerns about a method that was previously unused.

As previously stated regarding single-instrument ligation of the appendix, the aim was to ligate the appendiceal stump. However, in our study, there was no ligation procedure performed for closure on the specimen side. Ligation of the specimen side could further prolong the closure time. Moreover, not ligating the specimen side carries a potential risk of contamination. In our study, closure and transection on the specimen side were performed using a vessel sealing device. Pogorelić and colleagues, in their study, closed the appendiceal stump in 115 patients without using clips or sutures, employing a harmonic scalpel instead [22]. They also reported that closure of the appendiceal stump was safe. However, their patients were children.

In our study, the fact that the technique was performed by a single surgeon limits its generalizability and does not provide insight into the learning curve. However, a separate and dedicated study is needed to evaluate the learning curve of this technique. Among the limitations of this study are the absence of a cost analysis for the method in question and the fact that the study was conducted at a single center.

## Conclusion

In laparoscopic appendectomy, closure of the appendix root using a single instrument prolongs the operation time compared to clip closure. Ligature and clip closure methods are associated with similar complications. This ligation method, which has not been previously used to close the appendix stump, can be performed using inexpensive basic laparoscopic materials available in every operating room without the need for additional surgical instruments or incisions. Furthermore, the ligation closure method should be considered a viable option for surgeons when closing appendix roots that are too wide for clip application.

## Supplementary Information

Supplementary Material 1.

## Data Availability

Further details and supporting data can be obtained from the corresponding author upon request.

## Abbreviations

SPSS: The statistical package for the social sciences
RR: Relative Risk
AA: Acute appendicitis
APA: Acute phlegmonous appendicitis
HL: Hodges–Lehmann Estimator
